# Highly Cross-Reactive and Protective Influenza A Virus H3N2 Hemagglutinin- and Neuraminidase-Specific Human Monoclonal Antibodies

**DOI:** 10.1128/spectrum.04728-22

**Published:** 2023-06-15

**Authors:** Michael Piepenbrink, Fatai Oladunni, Aitor Nogales, Ahmed M. Khalil, Theresa Fitzgerald, Madhubanti Basu, Christopher Fucile, David J. Topham, Alexander F. Rosenberg, Luis Martinez-Sobrido, James J. Kobie

**Affiliations:** a Heersink School of Medicine, Infectious Diseases, University of Alabama at Birmingham, Birmingham, Alabama, USA; b Department of Medicine, Infectious Diseases Division, University of Rochester, Rochester, New York, USA; c Texas Biomedical Research Institute, San Antonio, Texas, USA; d Department of Microbiology and Immunology, University of Rochester Medical Center, School of Medicine and Dentistry, Rochester, New York, USA; e Department of Zoonotic Diseases, Faculty of Veterinary Medicine, Zagazig University, Zagazig, Egypt; f Heersink School of Medicine, Informatics Institute, University of Alabama at Birmingham, Birmingham, Alabama, USA; Institute of Microbiology Chinese Academy of Sciences

**Keywords:** B cell responses, hemagglutinin, immunization, influenza, monoclonal antibodies, neuraminidase, neutralizing antibodies

## Abstract

Due to antigenic drift and shift of influenza A viruses (IAV) and the tendency to elicit predominantly strain-specific antibodies, humanity remains susceptible to new strains of seasonal IAV and is at risk from viruses with pandemic potential for which limited or no immunity may exist. The genetic drift of H3N2 IAV is specifically pronounced, resulting in two distinct clades since 2014. Here, we demonstrate that immunization with a seasonal inactivated influenza vaccine (IIV) results in increased levels of H3N2 IAV-specific serum antibodies against hemagglutinin (HA) and neuraminidase (NA). Detailed analysis of the H3N2 B cell response indicated expansion of H3N2-specific peripheral blood plasmablasts 7 days after IIV immunization which expressed monoclonal antibodies (MAbs) with broad and potent antiviral activity against many H3N2 IAV strains as well as prophylactic and therapeutic activity in mice. These H3N2-specific B cell clonal lineages persisted in CD138^+^ long-lived bone marrow plasma cells. These results demonstrate that IIV-induced H3N2 human MAbs can protect and treat influenza virus infection *in vivo* and suggest that IIV can induce a subset of IAV H3N2-specific B cells with broad protective potential, a feature that warrants further study for universal influenza vaccine development.

**IMPORTANCE** Influenza A virus (IAV) infections continue to cause substantial morbidity and mortality despite the availability of seasonal vaccines. The extensive genetic variability in seasonal and potentially pandemic influenza strains necessitates new vaccine strategies that can induce universal protection by focusing the immune response on generating protective antibodies against conserved targets within the influenza virus hemagglutinin and neuraminidase proteins. We have demonstrated that seasonal immunization with inactivated influenza vaccine (IIV) stimulates H3N2-specific monoclonal antibodies in humans that are broad and potent in their neutralization of virus *in vitro*. These antibodies also provide protection from H3N2 IAV in a mouse model of infection. Furthermore, they persist in the bone marrow, where they are expressed by long-lived antibody-producing plasma cells. This significantly demonstrates that seasonal IIV can induce a subset of H3N2-specific B cells with broad protective potential, a process that if further studied and enhanced could aid in the development of a universal influenza vaccine.

## INTRODUCTION

Although seasonal influenza vaccines are administered to protect against infection, vaccine effectiveness (VE) can vary substantially. Reasons for this include poor match of the vaccine strain to the circulating strain as a result of genetic drift and or shift (1), previous exposure through either influenza vaccination or natural infection that shifts the immune response away from mounting a response toward the new vaccine strain (2, 3), or changes in vaccine strains as a result of propagation in eggs (4, 5). Overall VE in the United States for the 2018-2019 influenza season was rather low, at only 29%, due in most part to the mismatch of reference strain (A/Singapore/INFIMH-16-0019/2016 H3N2) of the 3c.2a1 clade and the dominance of 3c.3a clade in the last half of the season (6). The VE against the 3c.3a viruses was estimated to be only 5%. In previous influenza seasons (2016-2017 and 2017-2018), egg-adapted H3N2 vaccine strains became antigenically distinct from circulating viruses due to egg-adapted strains losing a glycosylation site on the hemagglutinin (HA) (7). Influenza virus H3N2 HA recently gained an N-linked glycosylation site at antigenic site B (4) of current clade 3c.2a based on a K160T mutation. Likewise, neuraminidase (NA) has gained a glycosylation site at positions 245 to 247 that not only alters accessibility of the NA active site to neutralizing antibodies but also reduces its enzymatic activity (8). Generally, current influenza A virus (IAV) H3N2 strains undergo more dramatic genetic drift than pandemic 2009 H1N1 (H1N1pdm09) and influenza B virus (IBV) strains (17 and 5 to 6 times higher, respectively [7]). With these particular issues, it is more important than ever to identify additional therapeutics.

H3N2 IAV first emerged in humans in 1968 as a result of recombination of circulating H2N2 with avian viruses (9). These avian viruses contributed the surface glycoproteins and PB1 to this pandemic strain (10) that killed up to 1 million people globally between 1968 and 1969. Infections with H3N2 IAV continue to account for high numbers of hospitalizations, with persons greater than 65 years of age with comorbidities at higher risk of complications (9, 11).

HA exists as a trimer on the influenza virus particle, made up of a globular head (HA1) and mostly conserved stem or stalk region (HA2) (12). In humans, HA preferentially binds to the α2,6-linked sialic acid (*N*-acetylneuraminic acid) to facilitate cellular membrane fusion (13). In contrast, NA is a homotetrameric surface viral protein that cleaves sialic acid, freeing the HA from mucus and allowing for the release of viral progeny (14). The active site of NA is highly conserved between IAV and IBV strains (15), making NA an excellent target for current antivirals and also for potential universal vaccine or therapeutic monoclonal antibody (MAb) development.

Here, we identify three H3-specific and three N2-specific human monoclonal antibodies (hMAbs) isolated from participants 1 week following vaccination with a seasonal inactivated influenza vaccine (IIV). Most of these hMAbs bind virus isolated over the last 5 decades and protect mice in prophylactic and therapeutic challenge experiments.

## RESULTS

### Seasonal IIV induces H3- and N2-specific hMAbs.

**FIG 1:**
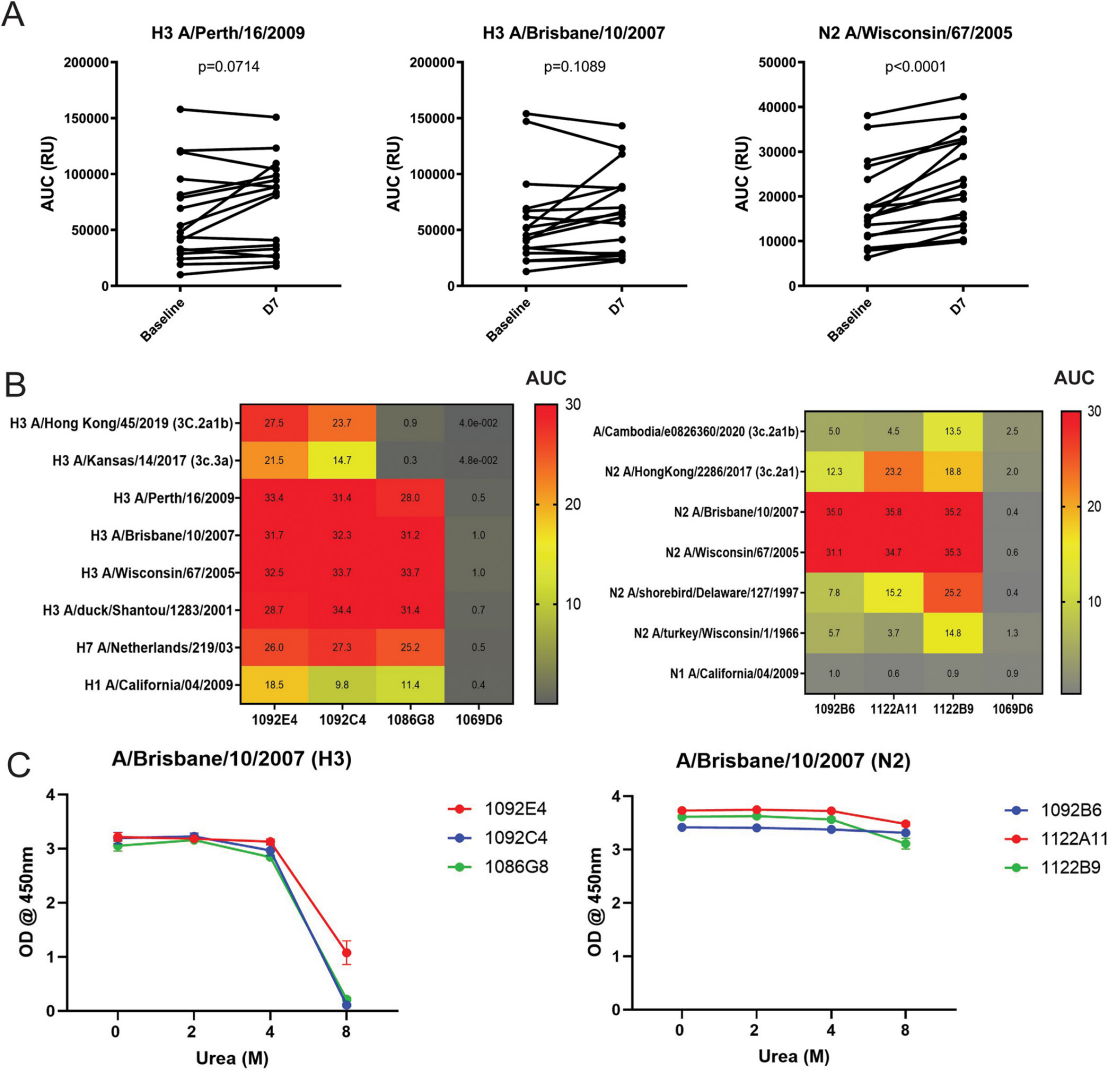
Induction of H3- and N2-specific plasma antibodies and breadth and avidity of binding of H3- and N2-specific antibodies. Peripheral blood was collected at baseline and at day 7 (D7) after immunization with IIV. H3- and N2-specific hMAbs were generated from plasmablasts following IIV immunization. (A) Plasma was serially diluted, and IgGs specific for H3 or N2 proteins were detected by ELISA; data for area under the curve (AUC) (*n* = 17 participants) are presented. (B) Increasing concentrations of hMAbs were tested for binding to the indicated HA and NA proteins by ELISA. 1069D6 was included in the assay as a nonspecific isotype IgG. Heat maps were generated based on AUC. (C) hMAbs were tested for avidity for H3 or N2 proteins of A/Brisbane/10/2007 H3N2 at 1 μg/ml in increasing concentrations of urea (0 to 8 M).

Peripheral blood was collected from 17 participants at baseline and 1 week after they were vaccinated with the 2014-2015 seasonal quadrivalent IIV. A trend toward increased plasma IgG specific for H3 A/Perth/16/2009 (*P* = 0.0714) and H3 A/Brisbane/10/2007 (*P* = 0.1089) and a significant induction of IgG specific for N2 A/Wisconsin/67/2005 (*P* < 0.0001) at 1 week after vaccination were observed (Fig. 1A). Recombinant human monoclonal antibodies (hMAbs) were isolated from peripheral blood plasmablasts from the participants with the highest plasma IgG response. The resulting best three H3-specific (1092C4, 1092E4, and 1086G8) and N2-specific (1092B6, 1122A11, and 1122B9) hMAbs with the broadest and most potent binding profiles were further characterized. The H3 hMAbs show a large breadth of reactivity against HA, including from both human and avian H3N2 influenza A virus (IAV) strains as well as the other group 2 IAV, A/Netherlands/219/03 H7N7 (Fig. 1B). The H3-specific hMAbs 1092C4 and 1092E4 also bound to HA from the more recent vaccine strains (A/Kansas/14/2017 H3N2 and A/Hong Kong/45/2019 H3N2). The N2-specific hMAbs were reactive to N2 of both A/Brisbane/10/2007 H3N2 and A/Wisconsin/67/2005 H3N2. The N2 1122B9 hMAb exhibited the broadest activity, including binding to N2 from earlier avian IAV isolates, along with binding to more recent A/Hong Kong/2286/2017 H3N2 and A/Cambodia/e0826360/2020 H3N2. The N2-specific 1122A11 hMAb also bound the N2 from A/Hong Kong/2286/2017 H3N2 and avian A/shorebird/Delaware/127/1997 H3N2 IAV strains. Avidity was measured by incubating bound hMAbs with increasing concentrations of urea. The H3-specific hMAbs did not exhibit any significant diminishment of binding in up to 4 M urea, but binding dramatically declined when urea was increased to 8 M (Fig. 1C). The binding of the N2-specific hMAbs was not significantly diminished by urea even at a concentration of 8 M. Together, these results indicate that the hMAbs have strong and broad binding to H3 or N2.

### Broad recognition of H3N2-infected cells.

**FIG 2:**
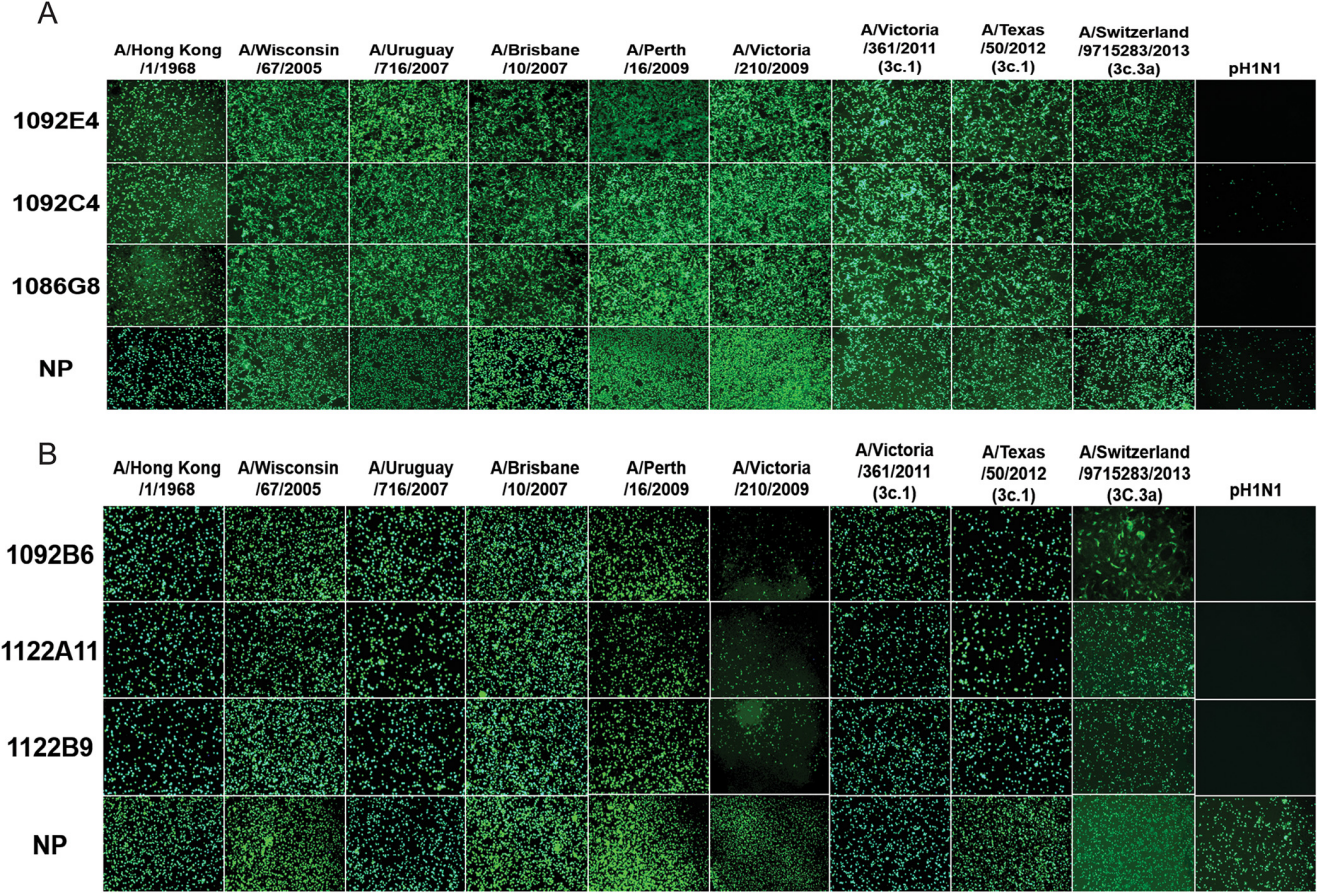
Characterization of H3 and N2 hMAbs by IFA. Confluent MDCK cells were infected with the indicated virus at an MOI of 0.1. At 16 h postinfection (hpi), cells were fixed and protein expression was evaluated by IFA using anti-HA-specific (A) or anti-NA-specific (B) hMAbs. An anti-NP polyclonal antibody was used as a positive control of infection, while pandemic A/California/04/2009 H1N1 (pH1N1) was included as a negative control. Images were taken using a 10× objective with a 500-ms exposure.

To define the ability of the hMAbs to recognize native HA and NA expressed during viral infection, Madin-Darby canine kidney (MDCK) cells were infected (multiplicity of infection [MOI] of 0.1) with human seasonal H3N2 IAVs and hMAb binding was evaluated by immunofluorescence assay (IFA). Viruses used ranged from A/Switzerland/9715283/2013 H3N2 to the historical A/Hong Kong/1/1968 H3N2. All H3 and N2 hMAbs had no to minimal binding to A/California/04/2009 H1N1-infected cells, which were included as a negative control. All three H3 hMAbs showed strong binding to all H3N2 IAV strains tested (Fig. 2A). The three N2 hMAbs also bound well to H3N2 IAV-infected MDCK cells, although binding to A/Victoria/210/2009 H3N2-infected cells was reduced, most likely because of reduced infection with this virus as indicated by nucleoprotein (NP) staining (Fig. 2B).

### Molecular characteristics of H3N2-specific hMAbs.

Two of the H3-specific hMAbs, 1092C4 and 1092E4, are clonally related, with their lineage utilizing VH1-18/VK3-20 (Table 1), and they differ only by three amino acids in the heavy chain and one in the light chain (see Fig. S1 in the supplemental material). The H3 hMAb 1086G8 utilizes VH3-23/VK3-15. These hMAbs resulted from moderate somatic hypermutation with 7.1 to 9.2% amino acid mutation from the germline for heavy chain variable regions and 6.3 to 9.5% for light chain variable regions. Heavy chain complementarity-determining region 3 (HCDR3) lengths ranged from 20 to 24 amino acids. The N2-specific hMAbs are all distinct lineages, also with moderate somatic hypermutation from the germline between 7.1 and 13.5% for heavy and 5.3 to 7.6% for light chain variable regions, respectively.

**TABLE 1:** Molecular characteristics of H3- and N2-specific hMAbs

hMAb	IgVH	IgDH	IgJH	HCDR3	Mutation (% NT/% AA)^*a*^	IgVL	IgJL	LCDR3	Mutation (% NT/% AA)^*a*^
H3 specific									
1092E4	IGHV1-18*01	IGHD3-3*01	IGHJ6*02	ARDHPQGVVILGSYYYGMDV	4.7/7.1	IGKV3-20*01	IGKJ1*01	QQSGSPRT	3.1/6.3
1092C4	IGHV1-18*01	IGHD3-3*01	IGHJ6*02	ARDLPQGVVILGSYYYGMDV	5.1/7.1	IGKV3-20*01	IGKJ1*01	QQSGSPRT	3.5/7.4
1086G8	IGHV3-23*01	IGHD3-9*01	IGHJ4*02	AKGVAPSHFNLLTGYYAGHYYFDF	5.8/9.2	IGKV3-15*01	IGKJ2*01	QQYNHWPPYT	4.2/9.5
N2 specific									
1092B6	IGHV1-24*01	IGHD3-10*01	IGHJ6*02	AAARRPIRGEYHYALDV	7.2/13.5	IGKV3-20*01	IGKJ1*01	HHYAKV	6.1/7.6
1122A11	IGHV1-46*01	IGHD3-22*01	IGHJ6*02	VRDLSHYNEVGHDRAYYYGMDI	6.1/11.2	IGLV3-1*01	IGLJ2*01	QAWDSSAVV	3.2/5.3
1122B9	IGHV3-23*04	IGHD2-2*01	IGHJ6*02	AKHTKSHYYSGMGV	3.7/7.1	IGKV1-33*01	IGKJ4*01	QQYDNLPLT	3.1/6.3

a NT, nucleotides; AA, amino acids.

### *In vitro* functional activity of H3N2-specific hMAbs.

The three H3-specific hMAbs were tested in hemagglutination inhibition (HAI) assay against virus strains isolated between 1968 and 2013 (Table 2). HAI activity varied substantially between H3N2 strains, with 1086G8 exhibiting activity only against A/Victoria/210/2009 H3N2, for which all hMAbs had a 50% inhibitory concentration (IC_50_) of <0.05 μg/mL. 1092C4 exhibited the broadest and most potent HAI activity, inhibiting 4/10 viruses at an IC_50_ of <10 μg/mL. All H3 and N2 hMAbs tested neutralized all the viruses at a 50% neutralization titer (NT_50_) of ≤50 μg/mL (Table 3), with H3 hMAb 1092C4 exhibiting the greatest breadth and most potent neutralizing activity, neutralizing 7/10 viruses at an NT_50_ of ≤3.13 μg/mL. This finding corroborates the HAI data, further alluding to the potent antiviral effect of 1092C4 *in vitro*. N2-specific hMAb 1122A11 was the broadest and most potent of the N2 hMAbs, neutralizing 5/10 viruses at an NT_50_ of ≤3.13 μg/mL.

**TABLE 2:** IC_50_s of the H3-specific hMAbs by hemagglutination inhibition assay

H3N2 virus	IC_50_ (μg/mL) of indicated H3 hMAb		
	1092E4	1092C4	1086G8
A/Hong Kong/1/1968	>100	>100	>100
A/Wisconsin/67/2005	6.25	6.25	>100
A/Uruguay/716/2007	50	12.5	>100
A/Brisbane/10/2007	3.13	<0.05	100
A/Perth/16/2009	>100	>100	>100
A/Victoria/210/2009	<0.05	<0.05	<0.05
A/Victoria/361/2011	6.25	0.8	100
A/Texas/50/2012	>100	>100	>100
A/Switzerland/9715293/2013	50	50	>100
A/Wyoming/3/2003	>100	100	>100

**TABLE 3:** IC_50_s of the H3 and N2 hMAbs by microneutralization assay

H3N2 virus	IC_50_ (μg/mL) of indicated MAb					
	H3 hMAb			N2 hMAb		
	1092E4	1092C4	1086G8	1092B6	1122A11	1122B9
A/Hong Kong/1/1968	6.25	6.25	1.6	6.25	3.13	ND^*a*^
A/Wisconsin/67/2005	0.2	1.6	3.13	50	25	ND
A/Uruguay/716/2007	0.1	0.2	0.4	25	0.2	ND
A/Brisbane/10/2007	0.1	0.1	0.4	3.13	6.25	ND
A/Perth/16/2009	3.13	3.13	6.25	6.25	0.2	ND
A/Victoria/210/2009	6.25	6.25	12.5	3.13	25	ND
A/Victoria/361/2011	0.1	0.1	0.2	25	1.6	ND
A/Texas/50/2012	6.25	6.25	6.25	6.25	3.13	ND
A/Switzerland/9715293/2013	6.25	0.78	12.5	50	25	ND
A/Wyoming/3/2003	3.13	1.6	0.78	0.4	6.25	ND

a ND, not done.

The ability of the N2-specific hMAbs to inhibit NA enzymatic activity was determined by enzyme-linked lectin assay (ELLA). Both 1092B6 and 1122A11 N2-specific hMAbs potently inhibited NA activity of all viruses at IC_50_s of ≤10 μg/mL, with 1122A11 exhibiting the greatest potency, with IC_50_s of <1 μg/mL for 7/8 viruses (Table 4). Again, this finding supports the earlier microneutralization data regarding the potent antiviral effect of 1122A11 in an *in vitro* setting. The ability of the hMAbs to inhibit the cleavage of a smaller substrate, 20-(4-NA-Star)-a-d-*N*-acetylneuraminic acid (NA-Star), was determined using recombinant N2 from A/Wisconsin/67/2005 H3N2 and A/Brisbane/10/2007 H3N2. Both 1122A11 and 1092B6 were able to inhibit NA activity efficiently (Table 5). 1122B9 did not inhibit NA activity even at 9 μg/mL, indicating that it binds to residues outside the NA active site. Because of this, 1122B9 was subsequently not studied further. Together, these results indicate that IIV induces H3 and N2 hMAbs with broad and potent antiviral activity against H3N2 IAV.

**TABLE 4:** IC_50_s of the N2 hMAbs by enzyme-linked lectin assay

H3N2 virus	IC_50_ (μg/mL) of indicated N2 hMAb		
	1092B6	1122A11	1122B9
A/Hong Kong/1/1968	0.63	0.31	ND
A/Wisconsin/67/2005	0.63	0.02	ND
A/Brisbane/10/2007	0.08	0.01	ND
A/Perth/16/2009	0.16	0.01	ND
A/Victoria/210/2009	0.04	0.01	ND
A/Texas/50/2012	0.08	0.01	ND
A/Switzerland/9715293/2013	1.3	0.02	ND
A/Wyoming/3/2003	0.63	10	ND

**TABLE 5:** IC_50_s of the N2 hMAbs by NA-Star

N2 protein^*a*^	IC_50_ (μg/mL) of indicated N2 hMAb		
	1092B6	1122A11	1122B9
A/Wisconsin/67/2005	1.74	2.05	>9
A/Brisbane/10/2007	1.99	1.44	>9

a NA proteins provided by BEI Resources.

### Persistence of H3 and N2 hMAb clonal lineage members in bone marrow.

**FIG 3:**
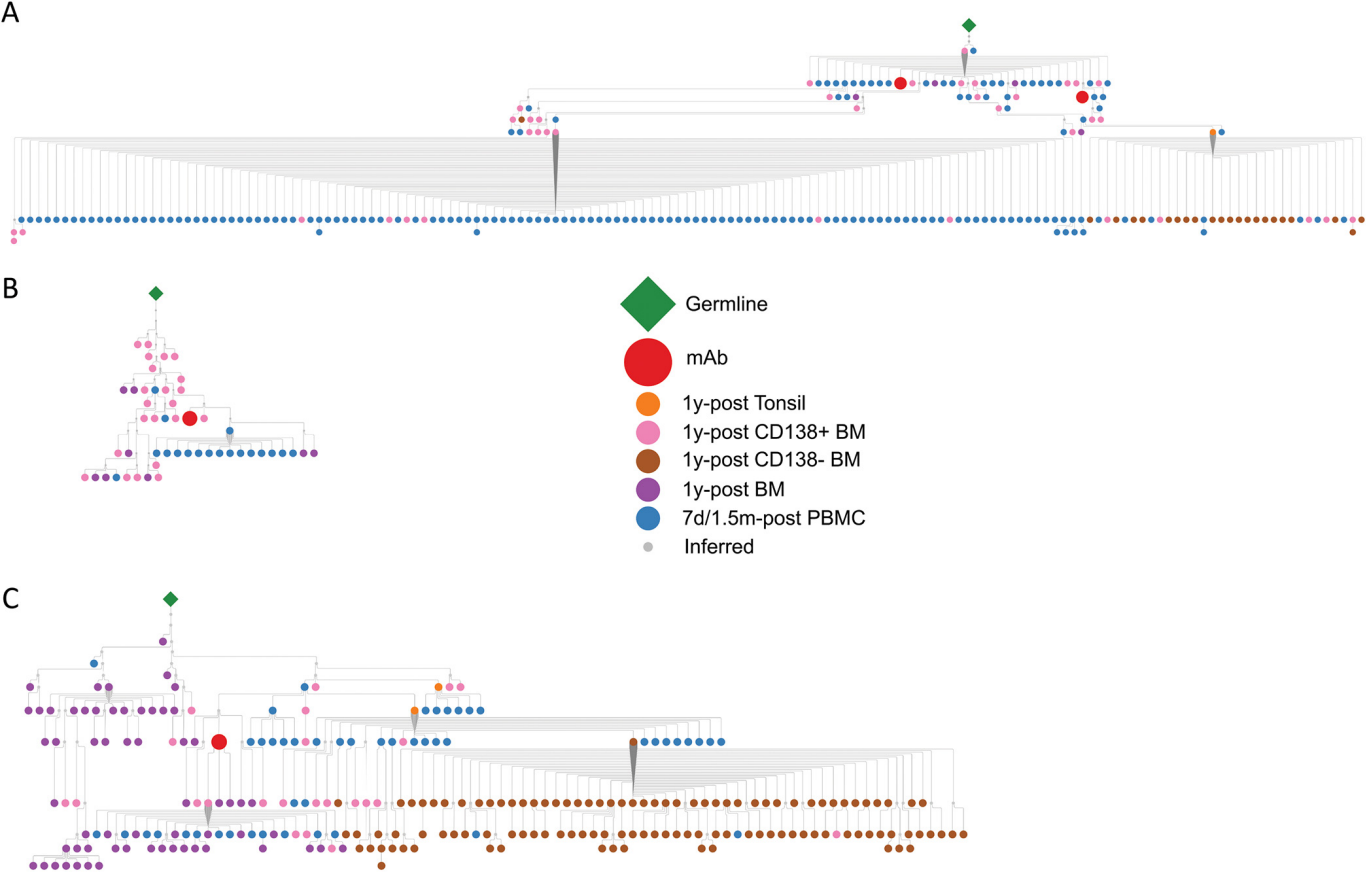
Clonal persistence of H3 and N2 hMAbs in bone marrow. 1092E4/C4 (A), 1122A11 (B), and 1092B6 (C) H3N2 hMAb lineage members were defined as exhibiting the same heavy chain V and J gene usage and HCDR3 lengths and ≥85% HCDR3 nucleotide similarity. Phylogenic analysis of these hMAb lineages was based on amino acid sequence. For simplification of viewing, only nodes representing five or more identical sequences are shown. Sequence analysis and assembly of lineage trees were performed using an in-house custom analysis pipeline as previously described (30, 36).

Targeted variable heavy (VH) chain immunoglobulin deep sequencing was utilized to help identify clonal lineage members of the best H3 and N2 hMAbs. The 1092E4/C4 lineage was highly expanded (Fig. 3A). Many lineage members were present in blood 7 days following vaccination and also in overlapping compartments including blood approximately 1.5 months later, and over 1 year later in bone marrow CD138^+^ long-lived plasma cells, as well as in the tonsil, suggesting long-term persistence. Members of the 1122A11 lineage were mostly identified in blood collected 7 days following vaccination, but several members of the lineage were also found in blood 1.5 months following IIV vaccination, as well as bone marrow CD138^+^ long-lived plasma cells over a year later (Fig. 3B). Lineage members of 1092B6 mostly existed in blood 7 days post-IIV vaccination, but members were also present in the bone marrow CD138^+^ long-lived plasma cells over a year after vaccination (Fig. 3C). These results indicate that H3 and N2 B cells with broad antiviral potential can persist in the long-lived plasma cell compartment of the bone marrow.

### Prophylactic and therapeutic activity of H3 and N2 hMAbs.

**FIG 4:**
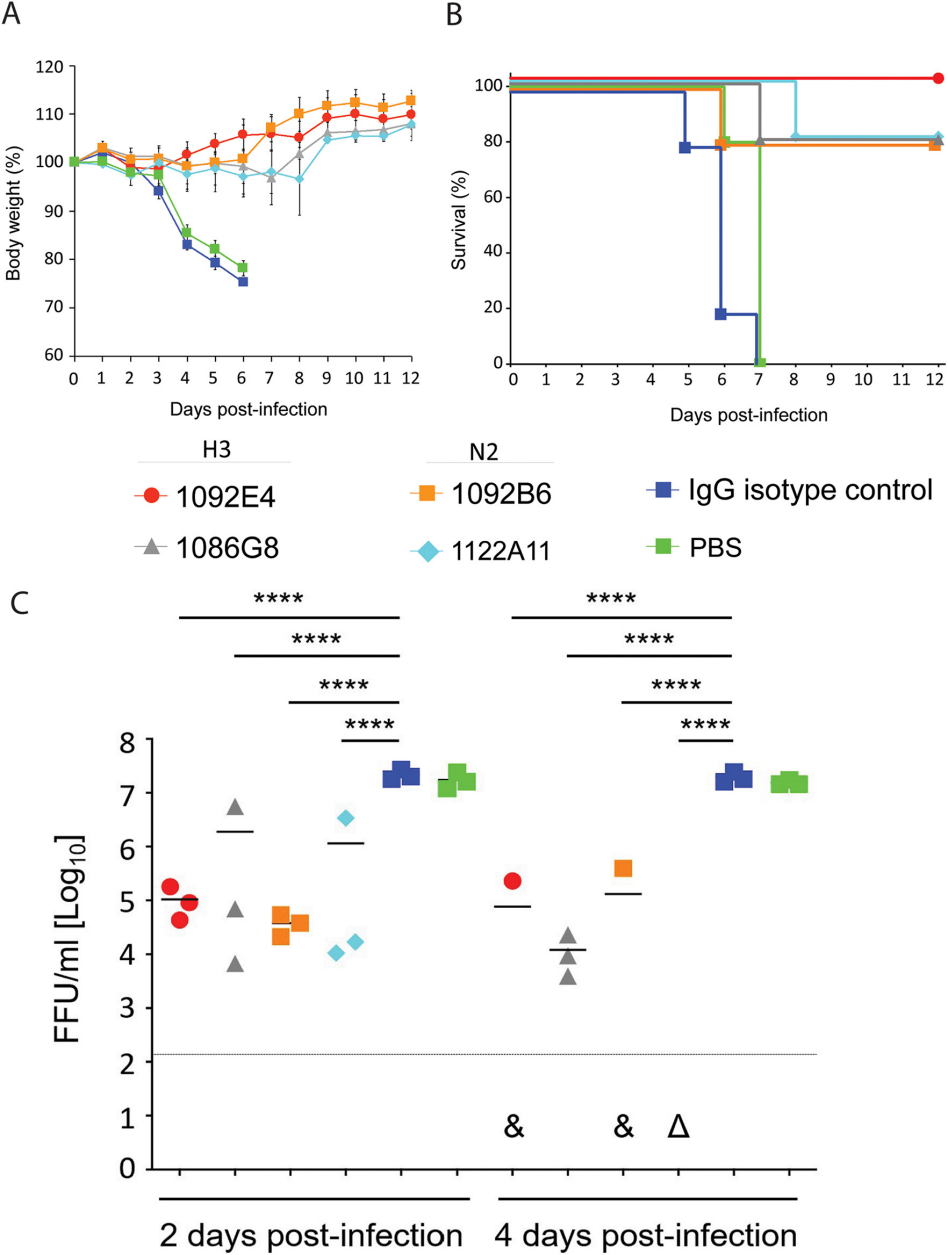
Prophylactic activity of H3 and N2 hMAbs in infected mice. Mice were given 20 mg/kg of indicated hMAbs or PBS, i.p., 24 h before infection. Mice were then challenged with 10× MLD_50_ of X-31 and monitored daily for 12 days postinfection (dpi) for body weight loss (A) and survival (B). To evaluate viral replication in the lungs (C), mice were sacrificed at 2 (*n* = 3) and 4 (*n* = 3) dpi and whole lungs were used to quantify viral titers by immunofocus assay with a starting dilution of 1:100. Each symbol represents an individual mouse. An ampersand indicates that virus was not detected in two of three mice in that specific group. A triangle indicates that none of the mice in a specific group had detectable virus. ****, significant differences (*P* < 0.0001 using one-way ANOVA and Tukey’s test. The long horizontal line indicates the limit of detection (LOD) of the assay (200 FFU). For measurements below the detection limit, 200 FFU was used in statistical analysis. For statistical analysis, actual measured viral titers were used, but for presentation, data were converted to log10 values.

Because recent strains of H3N2 virus do not replicate efficiently in mouse models, the historical reference strain, X-31 (a reassortant virus carrying the HA and NA genes of A/Hong Kong/1/1968 H3N2 in the background of A/Puerto Rico/8/34 H1N1) was used to determine the antiviral activity of H3 and N2 hMAbs in female C57BL/6 mice. To determine prophylactic activity, 20 mg/kg of body weight of 1092E4, 1086G8, 1122A11, or 1092B6 hMAb was administered intraperitoneally (i.p.). After 24 h, mice were challenged with 10× minimal lethal dose 50% (MLD_50_) of X-31. Previously, this dose produced 100% mortality in control C57BL/6 mice (16). All H3 and N2 hMAb-treated groups did not show weight loss, which was apparent in the phosphate-buffered saline (PBS)- and isotype control-treated mice (Fig. 4A). All mice treated with the H3-specific hMAb 1092E4 survived, whereas other H3 (1086G8) and N2 hMAbs (1122A11 and 1092B6) each resulted in an 80% survival rate, while all PBS- and isotype control-treated mice had to be euthanized by day 7 after challenge, upon reaching their endpoints for body weight loss (Fig. 4B). All H3 and N2 hMAbs resulted in significantly decreased viral titers in lungs of mice at days 2 and 4 postinfection compared to isotype control-treated mice (Fig. 4C). By day 4, differences in viral titers were more evident. Virus was below the detectable threshold in two of the three mice administered 1092E4 and 1092B6 and in all of the mice administered 1122A11. These data provide additional evidence of the dominant antiviral effects of 1092E4 and 1122A11 previously observed *in vitro*.

**FIG 5:**
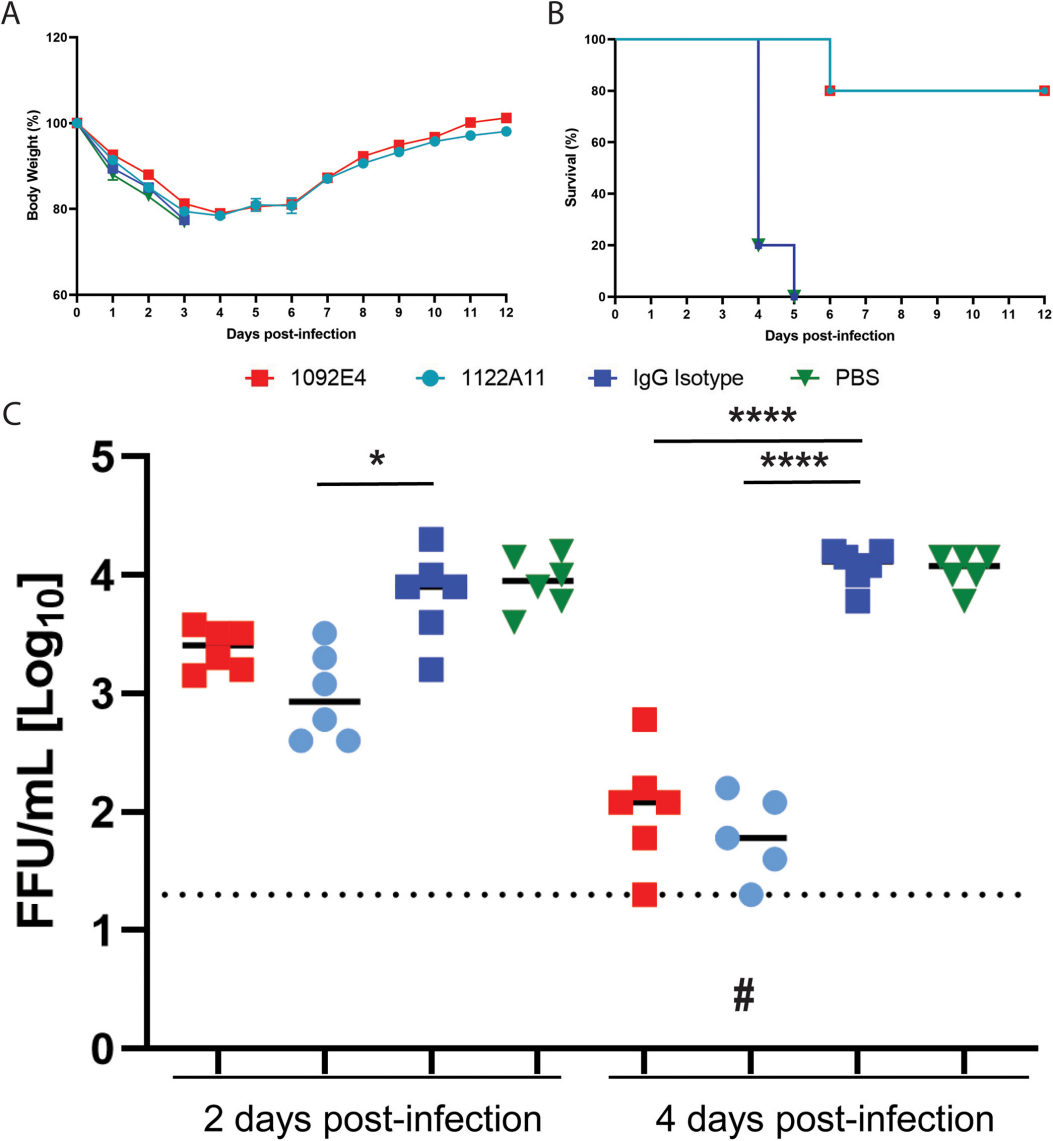
Prophylactic activity of 1092E4 and 1122A11 hMAbs in infected mice. Mice were given 10 mg/kg of indicated hMAbs or PBS, i.p., 24 h before infection. Mice were then challenged with 10× MLD_50_ of X-31 and monitored daily for 12 dpi for body weight loss (A) and survival (B). To evaluate viral replication in the lungs (C), mice were sacrificed at 2 (*n* = 6) and 4 (*n* = 6) dpi and whole lungs were used to quantify viral titers by immunofocus assay with a starting dilution of 1:100. Each symbol represents an individual mouse. A pound sign indicates that virus was not detected in one of six mice in that specific group. Significant differences (*, *P* < 0.05; ****, *P* < 0.0001) were determined using one-way ANOVA and Tukey’s test for multiple-comparison correction. The dotted line indicates the LOD of the assay (20 FFU). For measurements below the detection limit, 20 FFU was used in statistical analysis. For statistical analysis, actual measured viral titers were used, but for presentation, data were converted to log10 values.

Only the most potent H3- and N2-specific hMAbs (1092E4 and 1122A11, respectively) from the initial prophylactic experiment were tested in a follow-up, more refined prophylactic experiment with lower MAb dosages (10 mg/kg) and increased numbers of animals for greater resolution. Body weight declined for all infected mice through day 6 after infection (Fig. 5A), which resulted in all isotype control- and PBS-treated mice falling below the threshold of 75% of original body weight for humane euthanasia by day 5 postinfection (Fig. 5B). Only one of the mice from each of the 1092E4 and 1122A11 treatment groups fell below this level and needed to be euthanized on day 6 postinfection. All remaining mice administered either 1092E4 or 1122A11 recovered through the end of the 12-day observation period. Viral titers in the lungs 2 days postinfection were lower in mice treated with 10 mg/kg of 1092E4 (*P = *0.06) or 1122A11 (*P = *0.01) than for isotype control-treated mice. By day 4 postinfection, viral titers were greatly reduced (*P < *0.0001) in 1092E4- and 1122A11-treated mice compared to titers in mice administered the isotype control. These data provide additional evidence of the antiviral effects of 1092E4 and 1122A11 previously observed *in vitro*.

**FIG 6:**
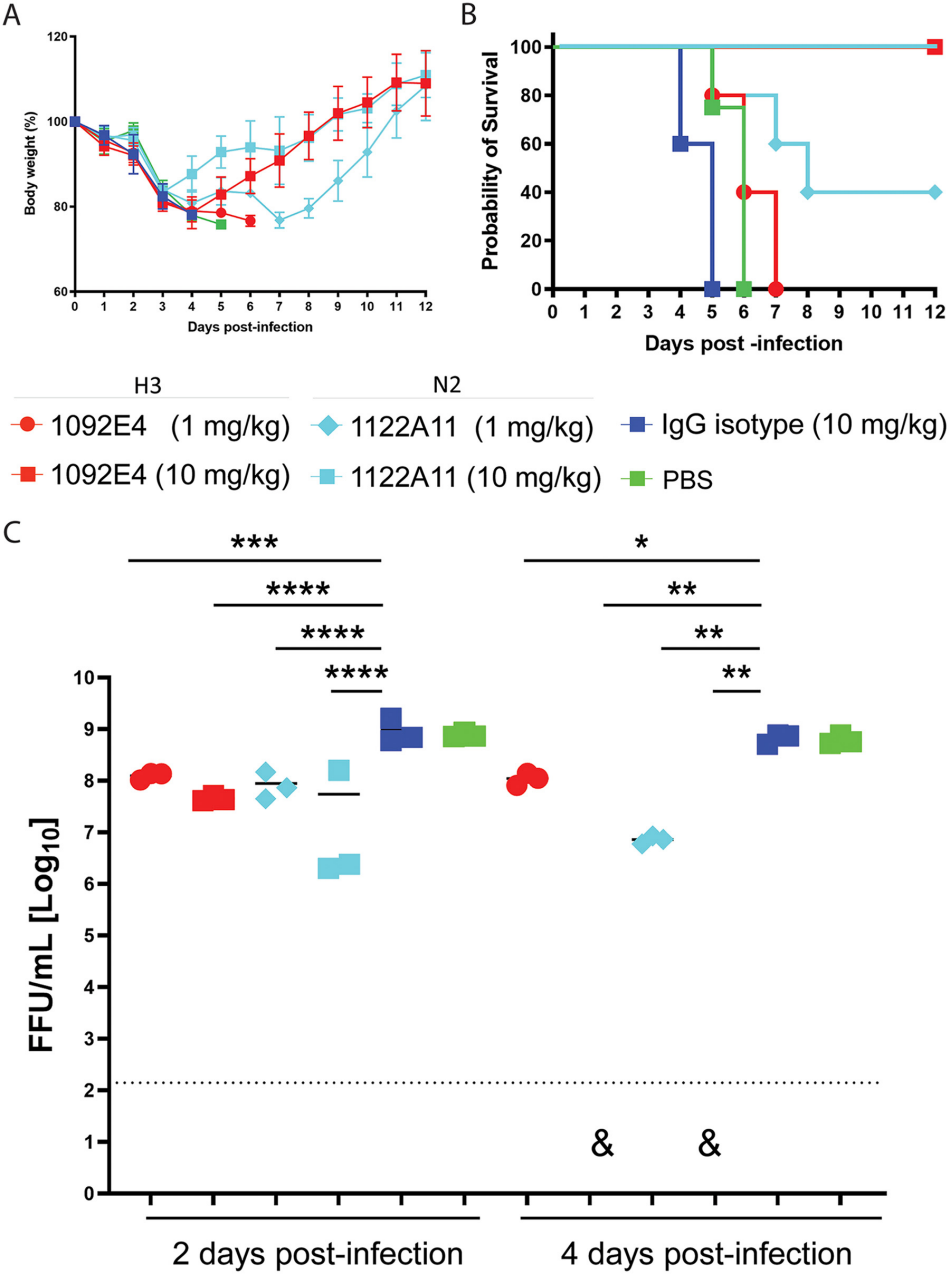
Therapeutic activity of H3 and N2 hMAbs in infected mice. Mice were infected with 10× MLD_50_ of X-31 and at 24 hpi were treated with indicated H3 (1092E4) or N2 (1122A11) hMAbs, or PBS, i.p. Mice were then monitored daily for 12 dpi for body weight loss (A) and (B) survival. To evaluate viral replication in the lungs (C), mice were sacrificed at 2 (*n* = 3) and 4 (*n* = 3) dpi and whole lungs were used to quantify viral titers by immunofocus assay with a starting dilution of 1:100. Each symbol represents an individual mouse. A triangle indicates that virus was not detected in all three mice in that specific group. Significant differences (*, *P* < 0.05; **, *P* < 0.01; ***, *P* < 0.001; ****, *P* < 0.0001) were determined using one-way ANOVA and Tukey’s test for multiple-comparison correction. The dotted line indicates the LOD of the assay (200 FFU). For measurements below the detection limit, 200 FFU was used in statistical analysis. For statistical analysis, actual measured viral titers were used, but for presentation, data were converted to log10 values.

We next tested 1092E4 and 1122A11 for therapeutic activity by administering the hMAbs 24 h after viral challenge. Body weight declined for all mice following infection (Fig. 6A) but started to recover by day 4 or 5 for animals that were administered 10 mg/kg of either 1092E4 or 1122A11. All the control animals treated with 10 mg/kg of isotype control or PBS had to be euthanized by day 6 after reaching their endpoints for body weight loss (Fig. 6B). All mice treated with 10 mg/kg of 1092E4 or 10 mg/kg of 1122A11 survived, and 40% of the mice treated with 1 mg/kg of 1122A11 survived. All mice treated with H3- and N2-specific hMAbs had significantly reduced viral loads in the lungs at days 2 and 4 compared to isotype control-treated mice (Fig. 6C). By day 4, virus was not detectable in lungs of mice treated with 10 mg/kg of 1092E4 or 1122A11. Together, these results indicated that the H3 1092E4 and N2 1122A11 hMAbs have potent prophylactic and therapeutic activity in influenza-challenged mice.

## DISCUSSION

Although vaccination is probably our best defense against influenza virus infection, vaccine effectiveness against strains that have mutated away from the ones used in the vaccine can leave individuals poorly protected (17). A lot of emphasis has been placed on the HA component of influenza vaccines. Unfortunately, most antibodies against HA tend to be against the highly variable globular head, providing only narrow protection against virus that is very similar to the vaccine strains (18, 19). The broad range of binding of 1092E4 (H3 specific) and 1122A11 (N2 specific) hMAbs by both enzyme-linked immunosorbent assay (ELISA) and IFA, as well as their neutralization activity, suggests that these hMAbs bind to highly conserved epitopes.

The receptor binding site (RBS) of HA is quite conserved among IAV strains. Often, hMAbs that bind this site have long HCDR3s (19 to 23 residues), including 019-10117 3C06 (20), 8F8 (21), and F045-092 (22). The three hMAbs described here have long HCDR3s ranging from 20 (1092C4 and E4) to 24 (1086G8) amino acid residues. F045-092 was shown to bind not only H3N2 viruses but also H1 and H13 and demonstrates viral neutralization against A/Okuda/1957 H2N2 and A/New Caledonia/20/1999 H1N1 (22). Our H3-specific hMAbs, particularly 1092E4, showed great breadth of binding to H3N2 viruses but also bound to H1 and H7 IAV by ELISA. Given the high level of homology between 1092E4 and 1092C4, further in-depth comparison of their *in vitro* and *in vivo* activities against H1 and H7 might provide additional insights regarding the molecular determinants of breadth. Unlike the Abs that have been shown to bind to the RBS, our hMAbs are not VH1-69 (019-10117 3C06 and F045-092) or VH3-33 (8F8) but are VH1-18 or VH3-23. Although this cross-reactivity is interesting, we have not completed the antibody/antigen complex crystal structure, which is a limitation of this study. As a result, we can only speculate on the specific epitopes.

Based on the NA inhibition assays (ELLA and NA-Star), both N2-specific 1092B6 and 1122A11 hMAbs interact with the NA active site. Some Abs that target the conserved NA active site have the potential for neutralizing all group 1 and group 2 IAV and IBV strains. Stadlbauer et al. (23) identified three Abs that show broad NA-Star inhibition activity, with IG01 inhibiting activity of all group 1 and group 2 IAV NAs and B/Victoria/2/87 lineage NA. Whether N2 hMAbs 1092B6 and 1122A11 have direct interaction with the NA active site or just inhibit the activity through steric hindrance still needs to be determined. Given that binding of these hMAbs to N1 of A/California/04/2009 H1N1 is lacking by ELISA (Fig. 1B), it is unlikely that antibody residues bind only the NA active site and probably bind other more variable residues outside the active site.

The virus used in mouse prophylactic and therapeutic experiments (X-31) is phylogenetically distant from the vaccine strain and other recent circulating strains. Still, the hMAbs tested provided prophylactic and therapeutic protection. Similarly, 84% of N2-reactive antibodies cloned following H3N2 infection provided protection to mice when administered at 5 mg/kg 2 h prior to viral challenge with the historical strain, A/Philippines/2/1982 H3N2 (X-79) (24). Chen et al. (24) showed that seven of eight of these hMAbs also protected mice from severe weight loss and provided for recovery when administered at 10 mg/kg 48 h following viral challenge. Likewise, the three NA-specific hMAbs used by Stadlbauer et al. (23) (IG04, IE01, and IG01) protected mice both prophylactically and therapeutically against A/Philippines/2/1982 H3N2 challenge when administered at 5 mg/kg. Weight loss was transient and viral titers were below detection on days 3 and 6 postinfection for animals treated with these hMAbs. In comparison, mice treated prophylactically with the N2-specific hMAb 1122A11 also showed no detectable virus on day 4 postinfection.

Identifying hMAbs that have broad neutralization capabilities against influenza viruses is desirable for potential therapeutics to use in cases in which induced immunity has not occurred (pandemic strain, vaccine/circulating strain mismatch, immunocompromised individuals, etc.) or when viral infection involves a drug-resistant strain. Several hMAbs against influenza virus have already undergone phase 1 and 2 clinical trials (summarized in reference 25). Understanding the epitopes that broadly protective, anti-influenza hMAbs use to neutralize virus should help inform future vaccine development. Expectantly, new vaccines would target highly conserved epitopes of HA and/or NA so that vaccine mismatch would become a dilemma of the past.

In conclusion, we have been able to identify several hMAbs that bind to H3 or N2 proteins with high avidity following vaccination with seasonal IIV. These hMAbs have broad recognition of H3N2 IAV strains. Two of the N2-specific hMAbs (1092B6 and 1122A11) disrupt NA activity as shown by both ELLA and NA-Star inhibition activity. Two H3 (1086G8 and 1092E4) and two N2 (1092B6 and 1122A11) hMAbs tested in a mouse model provided protection when administered before H3N2 infection. When used therapeutically, 10 mg/kg of either 1092E4 (H3) or 1122A11 (N2) administered 24 h after H3N2 infection protected all the infected mice. These results indicate that vaccination with seasonal IIV can expand B cells with the potential for broadly protective activity against H3N2 viruses.

## MATERIALS AND METHODS

### Subjects.

Peripheral blood was obtained from 17 healthy adult participants prior to, 7 days, and 1 month after receiving the 2014 to 2015 seasonal quadrivalent IIV (Sanofi Pasteur Fluzone; A/California/07/2009 X-179A [H1N1] pdm09, A/Texas/50/2012 X-223A [H3N2], B/Massachusetts/2/2012, and B/Brisbane/60/2008 viruses) as the standard of care at the University of Rochester Medical Center. A 50-mL volume of bone marrow aspirate was obtained from the posterior iliac crest. The subjects provided written informed consent. All procedures and methods were approved by the Research Subjects Review Board at the University of Rochester Medical Center (institutional review board [IRB] protocol 00000563), and all experiments were performed in accordance with relevant guidelines and regulations. Peripheral blood mononuclear cells (PBMC) and plasma were isolated using cell preparation tubes (CPT) (Becton, Dickinson, Franklin Lakes, NJ, USA).

### Cells and viruses.

Madin-Darby canine kidney (MDCK; ATCC CCL-34) and human embryonic kidney (HEK293T; ATCC CRL-11268) cells were grown in Dulbecco’s modified Eagle’s medium (DMEM; Mediatech, Inc.) that had been enriched with 5% fetal bovine serum (FBS) and 1% PSG (penicillin, 100 units/mL; streptomycin, 100 g/mL; l-glutamine, 2 mM) at 37°C in a 5% CO_2_ incubator (16).

Influenza A/Hong Kong/1/1968 H3N2 (NR-2620), A/Wisconsin/67/2005 H3N2 (NR-41800), A/Uruguay/716/2007 H3N2 (NR-42003), A/Brisbane/10/2007 H3N2 (NR-12283), A/Perth/16/2009 H3N2 (NR-41803), A/Victoria/210/2009 H3N2 (NR-44005), and A/Victoria/361/2011 H3N2 (NR-44022) were obtained from BEI Resources (Manassas, VA). Influenza A/Texas/50/2012 H3N2 (FR-1210) and A/Switzerland/9715283/2013 H3N2 (FR-1368) were obtained from Influenza Reagent Resource (IRR). Recombinant influenza A/California/4_NYICE_E3/2009 (pH1N1) and A/Wyoming/3/2003 H3N2 viruses have been previously described (26, 27). Viral stocks and titrations were conducted in MDCK cells at 37°C. For infections, virus stocks were diluted in phosphate-buffered saline (PBS) containing 0.3% bovine albumin (BA)–1% penicillin-streptomycin (PS). After viral infections, cells were maintained in postinfection (p.i.) DMEM containing 0.3% BA, 1% PSG, and 1 μg/mL of tosylsulfonyl phenylalanyl chloromethyl ketone (TPCK)-treated trypsin (Sigma) (28, 29). Viral titers were determined by immunofocus assay using MDCK cells as previously described (28–30). Briefly, confluent wells of MDCK cells (96-well format, 10^4^ cells/well, triplicates) were infected with 10-fold serial dilutions of homogenized lungs. At 8 to 12 h postinfection, cells were fixed and permeabilized (4% formaldehyde and 0.5% Triton X-100 in PBS) for 15 min at room temperature. After washing with PBS, cells were incubated in blocking solution (2.5% bovine serum albumin [BSA] in PBS) for 1 h at room temperature, washed with PBS, and incubated with 1 μg/mL of a monoclonal antibody against influenza A virus nucleoprotein (NP) (MAb HB65) for 1 h at 37°C. After 3 washings with PBS, cells were incubated with a fluorescein isothiocyanate (FITC)-conjugated rabbit anti-mouse IgG secondary antibody (Dako) for 1 h at 37°C. Cells were then visualized under a fluorescence microscope and NP-expressing cells were enumerated to determine the virus titers as fluorescence-forming units (FFU) per milliliter.

### Generation and screening of hMAbs.

Fresh PBMC collected 7 days after immunization were stained for flow cytometry as previously described (31). Plasmablasts (CD19^+^ IgD^−^ CD38^+^ CD27^+^) were subjected to direct single-cell sorting performed with a FACSAria cell sorter (BD Biosciences) and were placed into 96-well PCR plates containing 4 μL of lysis buffer as previously described (31). Plates were immediately frozen at −80°C after sorting until thawed for reverse transcription and nested PCR performed for IgH, Igλ, and Igκ variable gene transcripts as previously described (31, 32). Paired heavy and light chain genes were cloned into IgG1 expression vectors and were transfected into HEK293T cells, and culture supernatant was concentrated using 100,000-molecular-weight-cutoff (MWCO) Amicon Ultra centrifugal filters (Millipore-Sigma, Cork, Ireland), and IgG was captured and eluted from Magne protein A beads (Promega, Madison, WI) as previously described (31, 32). Immunoglobulin sequences were analyzed by IgBlast (https://www.ncbi.nlm.nih.gov/projects/igblast/) and IMGT/V-QUEST (https://www.imgt.org/IMGT_vquest/vquest) to determine which sequences should lead to productive immunoglobulin, to identify the germline V(D)J gene segments with the highest identity, and to scrutinize sequence properties.

### Binding characterization (ELISA and avidity).

ELISA plates (Nunc MaxiSorp; Thermo Fisher Scientific, Grand Island, NY) were coated with recombinant NA or HA proteins (BEI Resources, Manassas, VA) at 1 μg/mL, hMAbs or plasma was diluted in PBS, and binding was detected with horseradish peroxidase (HRP)-conjugated anti-human IgG (Jackson ImmunoResearch, West Grove, PA). Plasma was tested in 5-fold dilutions (1:100 to 1:62,500), and values for area under the curve (AUC) were determined. In selected ELISAs, increasing concentrations of urea were added to the ELISA plate and the plates were incubated for 15 min at room temperature prior to detection with anti-IgG-HRP to evaluate binding avidity.

### Immunofluorescence assay (IFA).

Confluent monolayers of MDCK cells (2 × 10^5^ cells/well, 24-well plate format) were mock infected or infected at a multiplicity of infection (MOI) of 0.1 with the indicated viruses. At 8 to 10 h p.i., cells were fixed with 4% paraformaldehyde (PFA) and permeabilized with 0.5% Triton X-100 in PBS for 15 min at room temperature. Cells were then incubated for 1 h at 37°C with 1 mg/mL of H3- or N2-specific hMAbs or with H1-specific hMAb KPF1 (31) as a control. Then, cells were incubated with FITC-conjugated secondary anti-human Ab (Dako) for 1 h at 37°C. Images were captured using a fluorescence microscope (Olympus IX81) and camera (QImaging; Retiga 2000R) with a 10× objective.

### Virus neutralization and fluorescence-based microneutralization assays.

Virus neutralization assays were performed with natural isolates or mCherry-expressing viruses as previously described (26, 29, 31, 33). Briefly, confluent monolayers of MDCK cells (5 × 10^4^ cells/well, 96-well plate format, quadruplicates) were infected with 100 to 200 FFU of the indicated viruses. After 1 h of viral adsorption, virus was removed, and cells were maintained at 33°C in p.i. medium supplemented with 1 μg/mL of TPCK-treated trypsin and 2-fold serial dilutions of the indicated hMAbs (starting concentration of 10 μg/mL). For the fluorescence-based microneutralization assays, at 48 to 72 h p.i., cell monolayers were washed with PBS prior to red fluorescence quantification using a fluorescence plate reader (DTX-880; Becton Dickinson). Fluorescence values of mCherry virus-infected cells in the absence of hMAb were used to calculate 100% viral infection. Cells in the absence of viral infection were used to calculate the fluorescence background. Natural virus neutralization assays were determined by crystal violet staining at 96 to 120 h p.i. The mean and standard deviation (SD) of neutralization and 50% inhibitory concentrations (IC_50_s) were determined with a sigmoidal dose-response curve (GraphPad Prism; v7.0).

### ELLA and NA-Star assay.

The ability of N2-specific hMAbs to inhibit the activity of the viral NA was measured using a standard enzyme-linked lectin assay (ELLA), as previously described (34, 35). Briefly, 2-fold serial dilutions of the hMAbs (starting concentration of 1 μg/mL) were preincubated with the indicated viruses at a predetermined concentration of virus for 2 h at room temperature in Dulbecco’s PBS (DPBS; Gibco) supplemented with 1% BSA. Virus-hMAb dilutions were added to 96-well plates coated with 50 μg/mL of fetuin (Sigma) and incubated for 18 h at 37°C. Then, plates were extensively washed with PBS containing 0.05% Tween 20 and incubated with HRP-coupled peanut lectin agglutinin (Sigma) in diluent buffer for 2 h at room temperature. After washing of the plates with PBS-Tween, the reactions were developed with 3,3′,5,5′-tetramethylbenzidine (TMB) substrate (BioLegend) for 15 to 20 min at room temperature, quenched with 2 N H_2_SO_4_, and read at 450 nm (Vmax kinetic microplate reader; Molecular Devices). The IC_50_ was determined with a sigmoidal dose-response curve (GraphPad Prism; v7.0).

The NA-Star influenza neuraminidase inhibitor resistance detection kit (Thermo Fisher Scientific) was used to test the inhibitory effect N2-specific hMAbs had on recombinant N2 protein activity as described by the product literature following the optimization of the assay for N2 protein (NR-49237, A/Wisconsin/67/2005; NR-43784, A/Brisbane/10/2007; BEI Resources). Briefly, the recombinant NA was diluted to 1 μg/mL in NA-Star assay buffer containing 100 μg/mL of BSA, and 25 μL was added per well except no-NA controls in the white assay plates. The hMAbs were serially 3-fold diluted in NA-Star assay buffer, with the highest concentration at 18 μg/mL. Then 25 μL of each dilution was added in triplicate to appropriate wells, mixed, and incubated at 37°C for 15 min. The diluted NA-Star substrate was then added to each well, and the plates were mixed and incubated for 30 min at room temperature. Finally, 60 μL per well of NA-Star accelerator was added and the chemiluminescent signal was read in a Cytation3 (BioTek, Winooski, VT).

### Prophylactic and therapeutic protective activities of H3- and N2-specific hMAbs in mice.

Female C57BL/6 mice (5 to 7 weeks of age) were purchased from the National Cancer Institute (NCI) and maintained in the animal care facility at the University of Rochester under specific-pathogen-free conditions. All animal protocols were approved by the University of Rochester Committee of Animal Resources and complied with the recommendations in the *Guide for the Care and Use of Laboratory Animals* of the National Research Council (36). For viral infections, mice were anesthetized intraperitoneally (i.p.) with 2,2,2-tribromoethanol (Avertin; 240 mg/kg of body weight) and were then inoculated intranasally (i.n.) with 10× MLD_50_ of influenza A/X-31 virus (H3N2; reassortant virus carrying the HA and NA genes of A/Hong Kong/1/1968 H3N2 in the background of influenza A/Puerto Rico/8/34 H1N1) in a final volume of 30 μL. To determine the prophylactic efficacy of the H3 and N2 hMAbs, at 24 h before infection, mice (*n* = 11) were subjected to i.p. administration of 20 mg/kg of the hMAbs, an IgG isotype control (BioXcell, Lebanon, NH), or PBS. This antibody concentration was based on our experience with other influenza-specific antibodies (31, 35). A second prophylactic experiment was completed at the Texas Biomedical Research Institute (San Antonio, TX) in the animal care facility under specific-pathogen-free conditions following animal protocol approval. For this study, the number of mice per treatment was increased to 17 and hMAb (1092E4, 1122A11, and isotype IgG) dosage was reduced to 10 mg/kg, 24 h before infection. For the study of therapeutic efficacy, at 24 h p.i., groups of mice (*n* = 11) were given i.p. injections of 10 or 1 mg/kg of either 1092E4 H3-specific or 1122A11 N2-specific hMAb. Mice administered either 10 mg/kg of IgG isotype antibody i.p. or PBS were included as controls. Viral replication was determined in the lungs of the infected mice at days 2 and 4 p.i. To determine the levels of replication, three or six mice from each group were euthanized by administration of a lethal dose (480 mg/kg of body weight) of 2,2,2-tribromoethanol and exsanguination. Lungs were then surgically extracted and homogenized. Virus titers (FFU per milliliter) were determined by immunofocus assay as indicated above (28, 29, 31). Geometric mean titers and data representation were performed using GraphPad Prism (v7.0).

### Deep-sequencing immunoglobulin repertoire analysis.

PBMC were isolated from whole blood collected into CPT as described above. In addition to the samples collected 7 days after immunization, PBMC were also isolated from blood samples collected more than 2 months prior to vaccination, 7 weeks after vaccination, and more than 15 months after vaccination. For the final blood sample, approximately 50 million PBMC were used to enrich for B cells by using biotinylated anti-CD3, anti-CD4, and anti-CD14 antibodies along with anti-biotin microbeads (Miltenyi Biotec, Auburn, CA) in a negative selection. Bone marrow aspirate was obtained at more than 12 months following the influenza vaccination, and mononuclear cells were isolated by floating the cells over Ficoll-Paque Plus medium (GE Healthcare BioSciences, Pittsburgh, PA). Approximately 40 million cells were then used with CD138 microbeads (Miltenyi Biotec) to isolate the CD138-positive fraction according to the manufacturer’s protocol. The entire positive fraction was lysed in RLT buffer (Qiagen, Hilden, Germany; catalog no. 79216) and stored at −80°C until RNA isolation could be performed. RNA was isolated from all samples using an RNeasy minikit (Qiagen), treated with DNase I (Turbo DNA-free kit; Invitrogen, Vilnius, Lithuania), and used to synthesize cDNA with a qScript cDNA synthesis kit (QuantaBio, Beverly, MA). The resulting cDNA was used in subsequent PCR using Platinum *Taq* high-fidelity polymerase (Invitrogen, Carlsbad, CA) as previously described (31). Targeted PCR was performed to try to detect lineage members of the cloned hMAbs by using forward primers specific for VH1-18, VH1-24, VH1-46, and VH3-23 (31). Gel-extracted PCR products were submitted to the University of Rochester Genomics Research Center, where Qubit fluorometric quantitation (Thermo Fisher) and Bioanalyzer (Agilent Technologies, Santa Clara, CA) sizing, quantitation, and quality control were performed prior to normalization to 2 nM and flow cell hybridization and cluster generation for a MiSeq system (Illumina, Inc., San Diego, CA). Paired-end reads (300 by 325 bp) were made. Sequence analysis and assembly of lineage trees were performed using an in-house custom analysis pipeline as previously described (31, 37). All sequences were aligned using IMGT.org/HighVquest (38). Lineage trees were generated by identifying the lineage (the cluster of sequences with identical VH, JH, and HCDR3 lengths and >85% HCDR3 nucleotide similarity) containing the corresponding hMAb sequence. Sequences within a lineage with single occurrences of particular VDJ nucleotide sequences (singletons) were removed, with the exception of singletons obtained from CD138 bone marrow samples. The resulting sequences were analyzed using Phylip’s protpars tool (v3.695) (39), turning on settings 1, 4, and 5. The output file was then parsed using in-house custom scripts, collapsing any duplicate inferred sequences into an individual node, and was visualized using Cytoscape (40) as we have previously reported (31, 35, 41).

### Statistical analysis.

Significance was determined using GraphPad Prism, v7.0. Paired *t* tests were applied for evaluation of the results of the serum binding antibody assessment. Initially, *in vivo* viral titers were subjected to analysis of covariance (ANCOVA) to test for effect of MAb treatment, day, and the MAb-by-day interaction. Both day and the MAb-by-day interaction were not significant (*P > *0.05), which allowed one-way analysis of variance (ANOVA) and Tukey’s test for multiple-comparison correction to be used to determine the statistical significance of the *in vivo* viral titers. For measurements below the limit of detection, either 20 or 200 FFU was assigned to those values based on the initial dilution of lung homogenate (1:10 or 1:100) used in the assay for statistical analysis.
