# Correction for Piepenbrink et al., “Highly Cross-Reactive and Protective Influenza A Virus H3N2 Hemagglutinin- and Neuraminidase-Specific Human Monoclonal Antibodies”

**DOI:** 10.1128/spectrum.03389-23

**Published:** 2024-01-30

**Authors:** Michael Piepenbrink, Fatai Oladunni, Aitor Nogales, Ahmed M. Khalil, Theresa Fitzgerald, Thomas A. Hilimire, Madhubanti Basu, Christopher Fucile, David J. Topham, Alexander F. Rosenberg, Luis Martinez-Sobrido, James J. Kobie

## AUTHOR CORRECTION

Volume 11, no. 4, e04728-22, 2023, https://doi.org/10.1128/spectrum.04728-22. The name of one author, Thomas A. Hilimire, was missing from the byline. The byline should appear as given in this Author Correction.

