# Supplementary material for: Highly Cross-Reactive and Protective Influenza A Virus H3N2 Hemagglutinin- and Neuraminidase-Specific Human Monoclonal Antibodies
Source: Microbiol Spectr. 2023 Jun 15;11(4):e04728-22. doi: 10.1128/spectrum.04728-22 (PMC10433997; doi:10.1128/spectrum.04728-22)
Supplement: Supplemental file 1 — Supplemental material. Download spectrum.04728-22-s0001.docx, DOCX file, 0.7 MB [file spectrum.04728-22-s0001.docx]

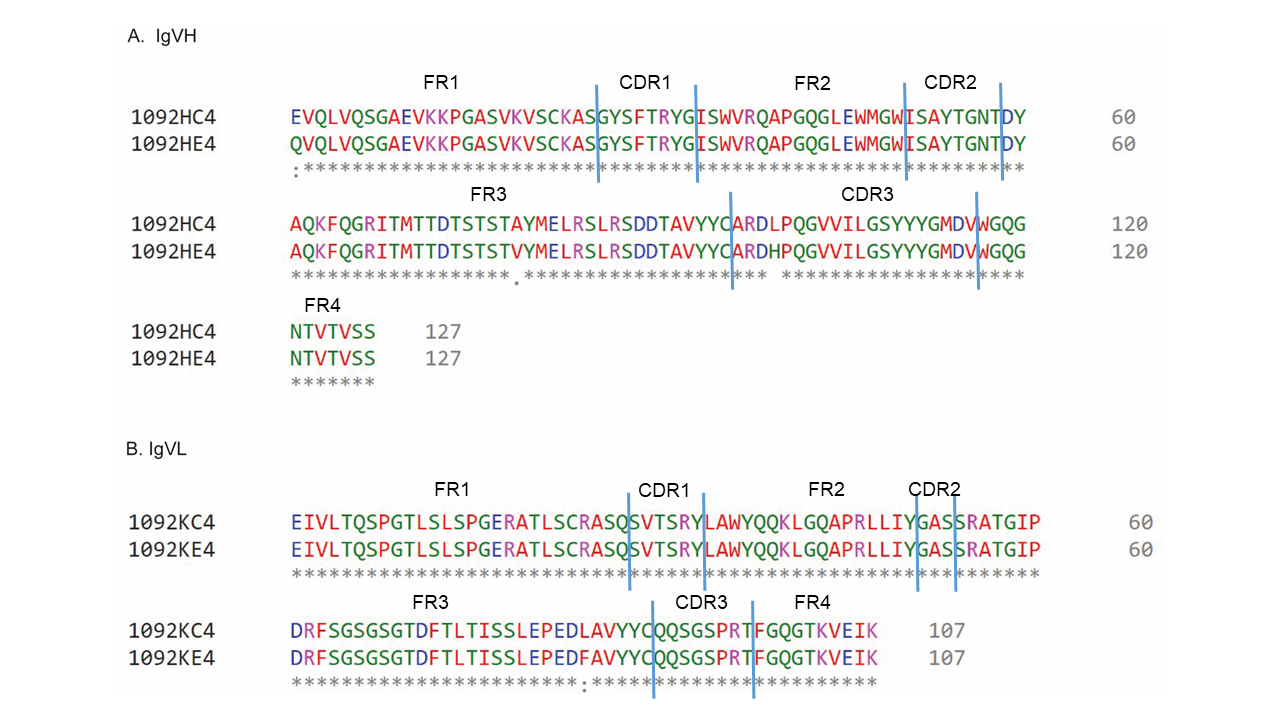


**Figure S1. Amino acid alignment of hMAbs 1092E4 and 1092C4.** The variable heavy (A) and variable kappa (B) chains were aligned using the amino acid sequences in Clustal Omega (EMBL-EBI). Identical amino acids are indicated below the alignment by *. Other or lack of a symbol show sites where different amino acids are present.
